# Cytomegalovirus-Specific T-Cell-Receptor-like Antibodies Target In Vivo-Infected Human Leukocytes Inducing Natural Killer Cell-Mediated Antibody-Dependent Cellular Cytotoxicity

**DOI:** 10.3390/ijms252312908

**Published:** 2024-11-30

**Authors:** Moritz Bewarder, Konstantinos Christofyllakis, Milena Petersen, Gerhard Held, Sigrun Smola, Gabi Carbon, Birgit Bette, Annika Link, Maximilian Kiefer, Joerg Thomas Bittenbring, Igor Age Kos, Vadim Lesan, Dominic Kaddu-Mulindwa, Lorenz Thurner, Frank Neumann

**Affiliations:** 1 Internal Medicine I, Saarland University Medical Center, 66421 Homburg, Germanybirgit.bette@uks.eu (B.B.);; 2 Internal Medicine I, Westpfalz-Klinikum Kaiserslautern, 67655 Kaiserslautern, Germany; 3 Institute of Virology, Saarland University Medical Center, 66421 Homburg, Germany; sigrun.smola@uks.eu; 4 Helmholtz Institute for Pharmaceutical Research Saarland (HIPS), Helmholtz Centre for Infection Research, Saarland University Campus, 66123 Saarbrucken, Germany

**Keywords:** cytomegalovirus, T-cell receptor (TCR)-like antibodies, human leukocyte antigen (HLA) alleles, stem cell transplantation, solid organ transplantation

## Abstract

Cytomegalovirus (CMV) reactivation after stem cell or solid organ transplantation remains a major cause of morbidity and mortality in this setting. T-cell receptor (TCR)-like antibodies bind to intracellular peptides presented in major histocompatibility complex (MHC) molecules on the cell surface and may have the potential to replace T-cell function in immunocompromised patients. Three previously selected CMV-specific, human leukocyte antigen (HLA)-restricted (HLA-A*0101, HLA-A*0201 and HLA-B*0702) Fab-antibodies (A6, C1 and C7) were produced as IgG antibodies with Fc optimization. All antibodies showed specific binding to CMV peptide-loaded tumor cell lines and primary fibroblasts expressing the corresponding MHC-I molecules, leading to specific target cell lysis after the addition of natural killer (NK) cells. When deployed in combination as an antibody pool against target cells expressing more than one matching HLA allele, cytotoxic effects were amplified accordingly. CMV-specific TCR-like antibodies were also able to mediate their cytotoxic effects through neutrophils, which is important considering the delayed recovery of NK cells after stem cell transplantation. When tested on patient blood obtained during CMV reactivation, CMV-specific antibodies were able to bind to and induce cytotoxic effects in lymphocytes. CMV-specific TCR-like antibodies may find application in patients with CMV reactivation or at risk of CMV reactivation. In contrast to previous HLA/peptide-directed therapeutic approaches, the concept of a TCR-like antibody repertoire covering more than one HLA allele would make this therapeutic format available to a much larger group of patients.

## 1. Introduction

The double-stranded DNA virus, human cytomegalovirus (CMV), infects and establishes latency in myeloid precursors and monocytes and infects and reactivates from macrophages and dendritic cells (DCs) [1,2]. Usually, primary CMV infection resolves quickly, and the virus is controlled by CD8+, CMV-specific T-cells [3,4,5].

Iatrogenic T-cell defects after allogeneic stem cell transplantation (HSCT) or solid organ transplantation (SOT), as well as acquired immunosuppression from human immunodeficiency virus (HIV) infections, can result in CMV reactivation with significant morbidity and mortality rates [6,7,8,9,10]. Strategies to reduce CMV-related complications after transplantation include either CMV monitoring with pre-emptive therapy or prophylactic therapy. For prophylaxis after HSCT, the CMV terminase inhibitor letermovir has been available for several years, while its safety and efficacy to prevent CMV infection after high-risk kidney transplantation has been shown just recently [11,12]. Universal prophylaxis with (val-) ganciclovir is recommended for patients after heart and lung transplantation [6,10,13]. For CMV-associated disease after HSCT or SOT, ganciclovir or valganciclovir are currently recommended as first-line therapies despite the risks of myelosuppression and the selection of drug-resistant strains [6,7,13]. Second-line therapeutic options include foscarnet and cidofovir, which were recently joined by the newly approved maribavir, a cytomegalovirus pUL97 kinase inhibitor [10,14,15]. Additional treatment options for CMV infection after allogeneic HSCT include the transfer of donor-derived or third-party cytomegalovirus-specific T-cells [16,17,18,19,20]. Nucleated cells display their intracellular proteome on the cell surface in major histocompatibility complexes class I (MHC I; human leukocyte antigen I (HLA I) in humans) [21,22,23]. After CMV infection of a cell, viral proteins are processed and presented on the cell surface via HLA class I complexes to CD8+ T-cells, leading to potent immune responses [24,25]. The efficacy of these T-cell responses seems to correlate directly with the number of detectable CMV-specific T-cells, although patient HLA status influences the number of CMV-specific T-cells that are needed for viral clearance [26]. CMV-specific antibodies, as part of the humoral immune response, play only a minor role in controlling CMV infection, and long-term immunity depends mostly on T-cells [27]. TCR-like antibodies (TCRL) bind specifically to peptides presented on MHC molecules and therefore have the potential to overcome immune defects that result from T-cell suppressive therapies. Broad application of TCRLs has been hampered by the polygenetic nature of HLA alleles, restricting those antibodies to a subset of patients with matching HLA alleles [28,29,30].

To circumvent this limitation, we developed a CMV-specific Fab antibody repertoire restricted to the alleles HLA-A*0101, HLA-A*0201 and HLA-B*0702 [31]. Respective Fabs showed binding to CMV-infected fibroblasts and were able to induce specific cytotoxicity in HLA-matched cell lines loaded with CMV peptides. CMV-specific TCRLs in Fab-format were expressed as IgG full-length antibodies with modified Fc regions and tested on peptide-loaded tumor cell lines and primary fibroblasts as well as fresh blood samples from CMV-infected patients and controls.

## 2. Results

### 2.1. Immunoglobulin G (IgG)-Format Cytomegalovirus (CMV)-Specific T-Cell Receptor-Like Antibodies (TCRLs) A6, C1 and C7

A6, C1 and C7 were expressed as Immunoglobulin G (IgG)1-format antibodies. When CMV-specific antibodies are used in a format other than IgG1, it is indicated separately. Correct molecular weight after expression was confirmed by Western blot. Binding to natural killer (NK) cells (via the Fc part of IgG1 antibodies) as well as CMV peptide-loaded lymphocytes expressing matching HLA alleles was confirmed by flow cytometry (Supplementary Materials Figure S1).

### 2.2. Increased Cytotoxicity of TCRLs After Fc Modification

The Fc-modified version of C1 induced lysis rates of 29% in fibroblasts loaded with pp65 peptide at 0.001 µg/mL and 48% at 0.01 µg/mL compared to only 5% at 0.01 µg/mL for the unmodified C1 version (Supplementary Materials Figure S2A). The unmodified variant of the B*0702-restricted TCRL (C7nm) induced significant antibody dependent cellular cytotoxicity (ADCC) only at high concentrations (7.5 µg/mL). After Fc modification, C7 started to induce specific lysis at concentrations of 0.01 µg/mL and reached lysing rates of 11% at 1 µg/mL (Supplementary Materials Figure S2B).

### 2.3. CMV-Specific Antibodies on CMV Peptide-Pulsed Tumor Cell Lines and Primary Fibroblasts

A6, C1 and C7 were tested on HLA-matched tumor cell lines for binding. All three TCRLs displayed strong binding to CMV peptide-pulsed cells (SK-Mel-23, T98G, A431) at 5 µg/mL but not after pulsing with DMSO only (Supplementary Materials Figure S3A).

A6, C1 and C7 were also tested on primary fibroblasts loaded with CMV peptides. A6, C1 and C7 were used at concentrations of 13.5 µg/mL, 5 µg/mL and 20 µg/mL and showed binding to CMV peptide-loaded primary fibroblasts with matching HLA alleles (Supplementary Materials Figure S3B). At an effector/target cell ratio of 10:1, CMV-specific antibodies started to induce specific lysis in peptide-loaded fibroblasts at concentrations of 0.01 µg/mL (specific lysis of A6, C1 and C7 at 4%, 5% and 3%). At concentrations of 1 µg/mL and a 10:1 effector/target ratio, A6, C1 and C7 induced 9%, 25% and 8.5% of specific lysis in CMV peptide-loaded fibroblasts (Supplementary Materials Figure S3C).

### 2.4. CMV-Specific Antibody-Mediated Phagocytosis by Neutrophils

Phagocytosis rates of MRC-5 cells loaded with CMV-peptide by polymorphonuclear leukocytes (PMNs) after the addition of C1 antibodies were assessed. MRC-5 cells are positive for HLA-A*0201 and epidermal growth factor receptor (EGFR) but negative for CD20. PMNs incubated with MCR5 cells loaded with the pp65 peptide aa495-503 but without antibody treatment resulted in phagocytosis rates of 4.4%, corresponding to the nonspecific background (Table 1, Supplementary Materials Figure S4A). After the addition of the CD20 antibody, rituximab (2 µg/mL), phagocytosis rates of PMNs against CMV peptide-loaded MRC-5 cells were 6.7% (negative control, Table 1). In contrast, the addition of cetuximab (2 µg/mL) led to a phagocytosis rate of 24.8% (positive control, Table 1). An additional positive control experiment was performed by adding the HLA-A*0201-specific antibody “B6” to MRC-5 cells loaded with the control peptide gp100 (aa637-645) and PMNs, leading to phagocytosis rates of 27.0% (Table 1). PMN-mediated phagocytosis rates of CMV peptide-pulsed MRC-5 cells after the addition of C1 ranged from 16.7% at 0.5 µg/mL to 22.7% at 2 µg/mL (Table 1, Supplementary Materials Figure S4B). When MRC-5 cells are loaded with the control peptide gp100 (aa637-645), C1 induces phagocytosis rates of 6.1% and 7.4% at 0.5 µg/mL and 2 µg/mL, respectively (Table 1).

### 2.5. Application of A6, C1 and C7 as CMV-Specific Antibody Pool

A*0201 and B*0702-positive and A*0101-negative lymphocytes were stained with the CMV-specific antibodies C1, C7 and A6. Antibody staining was performed either separately or in combination with an antibody mix. A6 (A*0101/CMV-restricted) showed no binding. C1 and C7 both demonstrated binding to CMV peptide-loaded lymphocytes. When lymphocytes were stained with all three antibodies as a pool, mean fluorescence intensity (MFI) increased, adding up to the MFIs generated by C1 and C7 (Supplementary Materials Figure S5A).

Cytotoxicity assays were performed accordingly. The addition of A6 did not result in specific lysis of CMV peptide-loaded lymphocytes. When used individually, the addition of C1 and C7 resulted in specific lysis rates of 40% and 35% at a concentration of 0.1 µg/mL, going up to 45% and 50% at 1 µg/mL, respectively. When all three CMV-specific antibodies were used in combination on CMV peptide-loaded lymphocytes, specific lysis rates increased to 53% at 0.1 µg/mL and 64% at 1 µg/mL (Supplementary Materials Figure S5B).

### 2.6. Specific Binding to Lymphocytes of CMV-Infected Patients by TCRLs A6, C1 and C7

Lymphocytes of nine patients after transplantation with active CMV infection and at least one matching HLA allele were stained with the CMV-specific antibodies A6, C1 and C7. A list of all nine patients is shown in Table 2.

Controls comprised of healthy donors without CMV infection and no matching HLA status, i.e., negative for HLA A*0101, A*0201 and B*0702 (Supplementary Materials Table S1 and Figure S6A); healthy donors without CMV infection with at least one matching HLA allele, i.e., either positive for HLA A*0101, A*0201 or B*0702 or a combination thereof (Supplementary Materials Table S2 and Figure S6B); and patients with CMV infection but no matching HLA status, i.e., negative for HLA A*0101, A*0201 and B*0702 (Supplementary Materials Table S3 and Figure S6C). A6, C1 and C7 showed no binding to lymphocytes of controls (Supplementary Materials Figure S6A–C).

**Figure 1:**
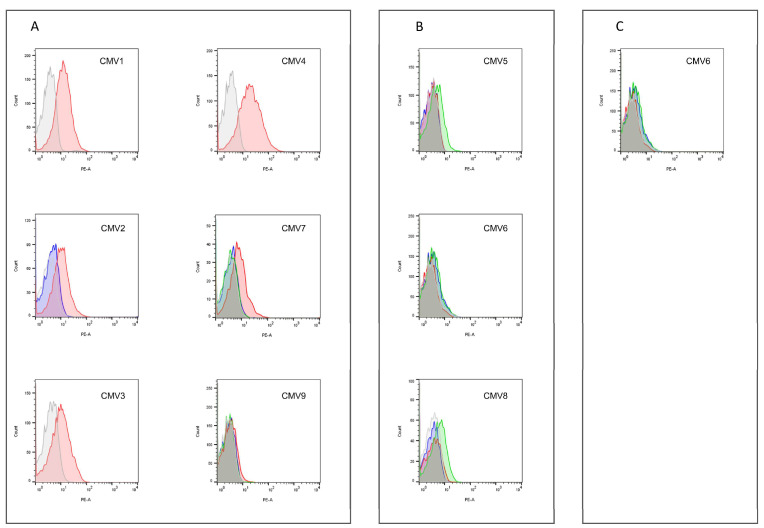
Cytomegalovirus (CMV)-specific T-cell receptor-like antibodies (TCRLs) bind to in vivo-infected lymphocytes. Peripheral blood samples of CMV-infected patients were stained with CMV-specific TCRLs C1 (**A**), A6 (**B**) and C7 (**C**). CMV viral loads were measured from peripheral blood on the same day and are shown in Table 2. C1 showed the most robust binding to in vivo-infected lymphocytes, but A6 and C7 were also able to bind to CMV peptide-presenting lymphocytes. The binding capacities of CMV-specific antibodies varied between all three antibodies and also between patients. These variations in binding capacities may result from differences in HLA complex presentation for each HLA allele and from differences in CMV load at the time the binding experiments were performed. Grey: negative control, Green: A6 antibody, Red: C1 antibody, Blue: C7 antibody.

C1 demonstrated robust binding to lymphocytes of all six CMV-infected HLA A*0201-positive patients (Figure 1A). Similarly, but with weaker intensity, A6 showed consistent binding to lymphocytes of three A*0101-positive patients with CMV infection (Figure 1B). One patient (CMV6) with CMV infection was positive for HLA B*0702. Staining of lymphocytes from this patient with C7 was weak but clearly detectable (Figure 1C). This patient was additionally positive for the HLA A*0101.

### 2.7. Correlation of CMV Viral Load with Staining Intensity of CMV-Specific Antibodies

**Figure 2:**
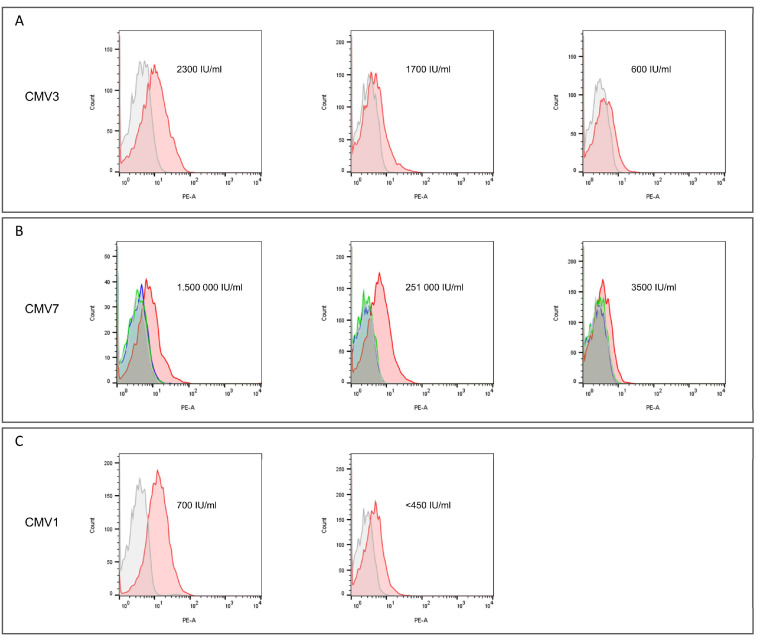
Staining intensity of CMV-specific TCRLs correlates with the viral load of patients. For three patients, longitudinal testing of CMV-specific antibodies was performed during the course of their CMV infections. In patient CMV3 (**A**), C1 showed strong binding to CMV-infected lymphocytes, with an infection load of 2300 IU/mL. In the same patient, staining intensity decreased during treatment and with dropping viral loads. In patient CMV7 (**B**), C1 showed strong binding to CMV-infected lymphocytes at both timepoints, with high viral loads (1.5 × 10^6^ and 251 × 10^3^ IU/mL). We believe that above a certain threshold of CMV copies, the loading limit of the existing MHC molecules is reached and a saturation effect occurs. This leads to the intensity of staining with CMV-specific TCRLs not increasing further. After effective treatment and a reduction in CMV copies to 3500 IU/mL, binding capacity was significantly reduced. Similar effects were observed in patient CMV1 (**C**), in whom the staining intensity of C1 correlated directly with measured CMV copies. The correlation of the staining intensity of CMV antibodies and CMV copy numbers was consistent within each patient, but not between different patients, e.g., 600 CMV IU/mL in patient CMV3 only resulted in very weak binding of C1, whereas strong binding was seen for C1 in patient CMV1 at 700 CMV IU/mL. Grey: negative control, Green: A6 antibody, Red: C1 antibody, Blue: C7 antibody.

For three of the patients, multiple measurements were obtained at different timepoints with different viral loads as measured in the patients’ peripheral blood. Staining intensities (measured by flow cytometry and given as MFI) varied in line with CMV viral loads in patient blood at the timepoint of staining experiments. Higher viral loads correlated positively with staining intensity. During the course of anti-CMV therapy, CMV DNA copies dropped, which was reflected by flow cytometry assays showing weaker binding of CMV-specific antibodies to CMV-infected lymphocytes (Figure 2A–C).

### 2.8. Specific Cytotoxicity of CMV-Specific TCRLs in PBMCs Obtained from CMV-Infected Patients

Whenever possible, we performed LDH release assays using PBMCs from CMV-infected patients as target cells and donor NK cells in order to assess whether the CMV-specific antibodies A6, C1 and C7 induce specific ADCC against CMV-infected target cells ex vivo. PBMCs of healthy volunteers with matching HLA alleles and no CMV infection served as controls.

**Figure 3:**
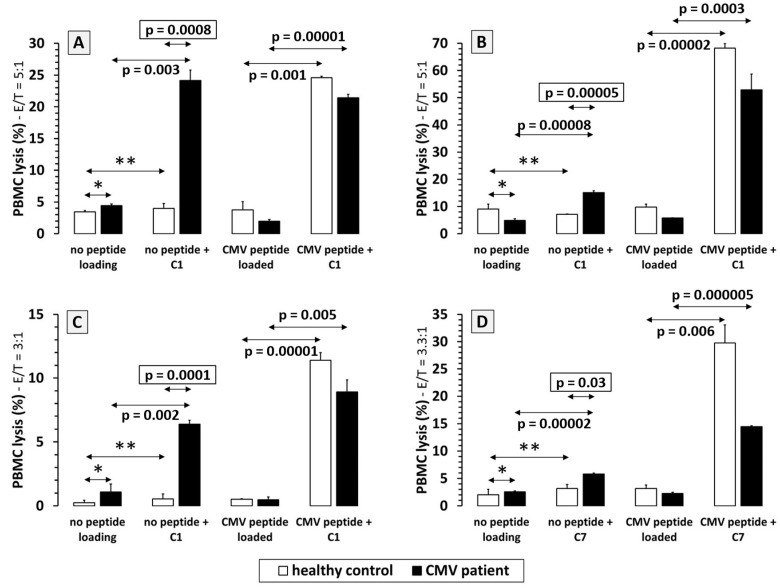
Natural killer (NK) cell-mediated antibody dependent cellular cytotoxicity (ADCC) of in vivo CMV-infected PBMCs. ADCC tests against HLA A*0201-expressing PBMC target cells of acute CMV-infected patients mediated by the A*0201/pp65(aa495-503)-specific IgG1 antibody “C1”. PBMCs from patients (**A**) CMV3, (**B**) CMV7 and (**C**) CMV1 (black columns). (**D**) ADCC assessment against HLA B*0702-expressing PBMC target cells of the acute CMV-infected patient CMV8 mediated by the B*0702/pp65(aa417-426)-specific IgG1 antibody “C7” (black columns). PBMCs of non-infected healthy donors were used as CMV-negative target cells (white columns). Patients and controls in (**A**–**C**) express the MHC-I subtype HLA A*0201, whereas the patient and control in (**D**) express the MHC-I subtype HLA B*0702. NK cells derived from the healthy controls were used as effector cells in the indicated E/T ratio (y-axis labeling) for experiments with patient lymphocytes (allogeneic) as well as for controls (autologous). Since no significantly different lysis (*) of native autologous PBMCs compared to allogeneic patient PBMCs was observed, an allo-specific ADCC by the NK cells of the respective healthy donor against patient PBMCs could be excluded. No significantly stronger ADCC (**) was induced against native PBMCs of healthy volunteers even when treated with the respective IgG1 construct (white columns: native/C1 and native/C7). In contrast, adding C1 or C7 to CMV-infected PBMCs resulted in a significant increase in NK cell-mediated ADCC (black columns: native/C1 and native/C7) compared to healthy PBMCs (native/C1 or C7: white vs. black column; framed *p*-values). As expected, loading PBMCs with CMV peptide induced strong ADCC after addition of the corresponding IgG construct (CMV/C1 or CMV/C7).

PBMCs of healthy donors and patients CMV3, CMV7 and CMV1 (HLA A*0201 positive) with CMV infection remained without peptide loading or were treated with C1. Donor NK cells induced spontaneous lysis of native PBMCs from 1% to 4%. When C1 was added to control PBMCs, no significant increase in specific lysis was observed. However, against CMV-infected PBMCs of patients CMV3, CMV7 and CMV1, we observed specific lysis rates of 24%, 16% and 7% after addition of C1 (Figure 3A–C).

HLA B*0702-positive PBMCs of patient CMV8 showed specific NK cell-mediated lysis rates of 6% after treatment with C7 compared to 2% lysis for the patients’ PBMCs not treated with C7 (Figure 3D).

## 3. Discussion

The intracellular proteome is presented on the cell surface as peptides in class I HLA molecules. Class I HLA alleles are polygenic in nature, with many thousands of reported allele variants [21,22,23,32]. HLA–peptide complexes on tumor cells or virus-infected cells represent attractive, highly specific targets for therapeutic approaches. These include TCRs and TCRLs with derivative immunotoxins and bi-specific T-cell engagers, adoptive cell transfer of TCR-engineered T-cells and CAR-T cells incorporating T-cell-receptor-like antibodies as receptors [28,32,33,34]. The FDA and EMA approval of the anti-gp100/HLA A*0201 TCR-based T-cell engager tebentafusp in 2022 shows the potential of HLA/peptide-directed therapies for clinical application. In patients with unresectable metastatic uveal melanoma, treatment with tebentafusp results in improved overall survival compared to immune- or chemotherapies [35].

With regard to CMV infection after stem cell transplantation, several HLA/peptide-directed strategies have been developed. Most approaches use donor-derived T-cells after ex vivo stimulation with CMV antigens [19,36,37,38]. More recently, CMV-specific T-cells generated from healthy donors were used for application in patients with at least one shared HLA class I allele [39]. Nevertheless, the routine use of CMV-specific T-cells remains complex and labor-intensive. In order to allow for a broader application of HLA/peptide-directed therapies, therapeutics should cover more than one HLA allele [39].

TCRLs share similar obstacles and, up to now, there are no TCRLs in clinical use. Establishing three CMV-specific antibodies restricted to the HLA alleles A*0101, A*0201 and B*0702, we aimed to overcome the main drawback of HLA/peptide-directed therapies by extending their applicability to a larger proportion of patients. The TCRLs described in this study might be useful in a therapeutic or prophylactic setting in patients after solid-organ or stem cell transplantation.

A6, C1 and C7 are CMV-specific TCRLs and have previously been tested as Fab-format antibodies. To advance the translational development of these antibodies, A6, C1 and C7 were now produced as IgG antibodies with modified Fc regions to increase ADCC. After stem cell transplantation, NK cell recovery is slow [40], raising the question of whether CMV-specific TCRLs can be effective in this setting since they are thought to rely on NK cells as effector cells. PMNs recover in a timely manner after stem cell transplantation, starting with engraftment approximately two to three weeks after stem cell transfusion, depending on the stem cell source and lymphodepletion regimen [41]. We therefore investigated and confirmed the potential of CMV-specific TCRLs to convey PMN-mediated phagocytosis. These results support the hypothesis that CMV-specific TCRLs may also be effective even if NK cells have not yet recovered or are suppressed in function by immunosuppressive therapies.

CMV-infected cells present CMV peptides in all HLA molecules transcribed and translated from their given HLA class I alleles. For patients with more than one matching HLA allele, the combination of two or three CMV-specific TCRLs may have the advantage of more potent ADCC. To explore the effects of the application of an antibody mix, we loaded donor PBMCs with a CMV peptide mix and performed staining and cytotoxicity assays using A6, C1 and C7 either separately or in combination as an antibody pool. The application of an antibody mix showed promising results as staining intensity and cytotoxicity of HLA-matching antibodies added up, while non-matching antibodies did not bind to CMV peptide-loaded PBMCs and did not exert relevant cytotoxicity.

Furthermore, the CMV-specific TCRLs A6, C1 and C7 were tested on peripheral blood of patients with CMV reactivation. Tetramerized Fabs were used as staining intensities with IgG antibodies were not sufficient for assessment by flow cytometry. As expected, most patients with CMV infection available for analysis expressed A*0201 (n = 6). Three patients with CMV infection were positive for A*0101, and only one was positive for B*0702. Despite significant variations in the CMV DNA load at the assessment timepoints, we were able to demonstrate robust and highly specific staining of CMV-infected lymphocytes (Figure 1). Lymphocytes of donors with different HLA alleles or without CMV infection remained negative upon staining with A6, C1 and C7. Interestingly, we observed a correlation between flow cytometric staining intensity and CMV DNA load, with more intense staining at timepoints with higher viral loads. The correlation between CMV copy numbers and flow cytometry staining intensity was consistent for each patient but did not hold up between patients. For example, one patient with 600 CMV IU/mL showed weak staining with C1, while another patient showed strong staining when 700 CMV IU/mL was detected. The correlation also seemed to be subjected to a ceiling effect, as no differences in staining intensity were observed for high (251 × 10^3^ IU/mL) and very high (1.5 × 10^6^ IU/mL) viral loads (Figure 2).

To demonstrate the efficacy of CMV-specific IgG antibodies, we performed cytotoxicity experiments using PBMCs from four CMV-infected patients (3 HLA A*0201-positive, 1 HLA B*0702-positive) as target cells. C1 and C7 showed highly specific ADCC when used against in vivo CMV-infected PBMCs ranging from 6% to 24%, but not against control PBMCs. It can be argued that the observed specific lysis rates are moderate, but the clinical setting in which CMV-specific antibodies might be useful and the optimal timepoint of antibody application are only speculative. In our opinion, this approach would be most effective as prophylactic treatment in patients with matching HLA alleles and who are at high risk of CMV reactivation.

Despite these findings, our study has several limitations. While we were able to show the binding of all three CMV-specific antibodies to in vivo-infected lymphocytes, ADCC experiments were only conducted for C1 and 7 but not for the A*0101-specific CMV antibody A6. This is due to the high complexity and long preparation time of ADCC tests in combination with the fact that the HLA allele A*0101 is relatively rare. Additionally, the number of patient samples, especially of HLA B*0702 and A*0101-positive samples, was quite low. Since HLA A and B alleles are downregulated during CMV infection, limiting the presentation of CMV peptides to T-cells and TCR-like antibodies, HLA-C-directed antibodies would be of great value [42]. Up to now, we have not been able to identify highly specific HLA-C-restricted CMV antibodies, defining the aim of future studies.

## 4. Material and Methods

### 4.1. Cells and Cell Culture

Punch biopsies of the skin were obtained using standard procedures. Biopsies were cut into small pieces and placed in Dulbecco’s Modified Eagle Medium (DMEM) with the addition of 10% Fetal Bovine Serum (FBS) and 1% Non-essential Amino Acids. After fibroblast cultures reached confluence, cells were passaged. Fibroblasts were passaged up to 15 times at a 1:3 to 1:5 ratio until they were discarded. For flow cytometry staining experiments, whole blood (EDTA tubes, SARSTEDT AG & Co. KG, Nuembrecht, Gemany) samples from patients and healthy controls were used. PBMCs were obtained from the EDTA blood of patients and healthy controls by density centrifugation on Ficoll-Hypaque as described previously. MRC-5 cells (ATCC number CCL-171) and T98G (ATCC number CRL-1690) were bought from the American Type Culture Collection (ATCC, Manassas, VA, USA) and cultured in Eagle’s Minimum Essential Medium (EMEM) supplemented with 10% FBS, 2mM glutamine, 100 U penicillin and 100 µg/mL streptomycin. SK-Mel 23 and A431 cell lines were kindly provided by Claudia Pfoehler (Saarland University Medical School, Homburg/Saar, Germany) and cultured in RPMI 1640 medium and DMEM, respectively. Both cell lines were supplemented with 10% FBS, 2 mM glutamine, 100 U penicillin and 100 µg/mL streptomycin (media and medium additives from PAN-Biotech GmbH, Aidenbach, Germany).

### 4.2. Isolation of NK Cells

Peripheral Blood Mononuclear Cells (PBMCs) were obtained by density gradient centrifugation from the blood of test subjects. Thereafter, NK cells were isolated from PBMCs by magnetic depletion of all non-NK cells using the CD56+/CD16+ human NK Cell Isolation Kit (Miltenyi Biotech GmbH, Bergisch Gladbach, Germany) according to the manufacturer`s instructions. NK cells were isolated one day before ADCC assays without additional activation. The viability of NK cells after isolation averaged 99% and the proportion of CD16+ cells was between 90% and 98%, as assessed by flow cytometry using the corresponding antibodies (Miltenyi).

### 4.3. Determination of HLA Status in Fibroblast and Lymphocyte Donors

The HLA status of patients was gathered from hospital data files. HLA status of healthy controls was obtained from data records of the German bone marrow donor registry (DKMS), where available, or by flow cytometry-based HLA typing using in-house HLA-specific antibodies.

### 4.4. Expression and Tetramerization of CMV-Specific Fabs

CMV-specific Fab antibodies were expressed as described previously [43,44]. For biotinylation, an AviTag sequence was attached to the C-terminal end of the heavy chain. The pCES1 vector containing the Fab sequence was transformed into bacteria of the *E. coli* strain AVB101, which contains an IPTG-inducible pACYC184 plasmid with the BirA gene. At the time of induction, biotin was added at a concentration of 50 μM [45]. R-phycoerythrin (R-PE)-conjugated streptavidin was added to Fab monomers in a molecular ratio of 1:4. Streptavidin PE was divided into 10 aliquots and added to Fab monomers at room temperature every 10 min [46].

### 4.5. Generation of CMV-Specific IgG TCRLs Restricted to HLA Alleles A*0201, A*0101 and B*0702

Variable regions of CMV-specific TCR-like Fab antibodies were cloned into a pSFI expression vector containing heavy and light chain constant regions of an IgG1 antibody via restriction enzymes. Full-length antibodies were produced by transfection into HEK293 cells for expression and purified via an integrated flag-tag.

### 4.6. Fc Modification of A6, C1 and C7

To increase ADCC activity, three amino acids (S239D, A330L, I332E) of the CH2 region of IgG1 CMV-specific antibodies were modified by mutagenesis using the QuikChange II Site-Directed Mutagenesis Kit (Agilent Technologies, Santa Clara, CA, USA) [47,48,49].

### 4.7. Peptide Pulsing of Tumor Cell Lines, Fibroblasts and PBMC

MRC-5, SK-Mel 23, T98G and A431 cell lines, as well as primary fibroblasts and lymphocytes of HLA-typed donors, were used as target cells. Peptide pulsing was performed as follows: 100 µL of EDTA blood was washed twice with PBS and incubated for 2 h at 37 °C in serum-free RPMI containing 20 µg/mL of peptide. Tumor cell lines and fibroblasts were pulsed overnight with 50 µg/mL of peptide in serum-free medium. The CMV peptides and control peptides used are shown in Table 3 and Table 4.

### 4.8. Isolation of Polymorphonuclear Neutrophils (PMNs) and Phagocytosis Assays

PMNs were isolated from the peripheral blood of healthy donors using two-phase density gradient centrifugation. For this, Pancoll (density of 1.077 g/mL, PAN-Biotec, Aidenbach, Germany) and Histopaque (density of 1.119 g/mL, Merck KGaA, Darmstadt, Germany) were used in the first step. PMNs were further purified using MACS according to the supplier’s protocol (Miltenyi) and using the following microbeads: anti-CD3, anti-CD56, anti-CD235a and anti-HLA-DR. The number and purity of isolated PMNs were determined by flow cytometry via CD66b expression.

To determine the activity of PMN-driven antibody-dependent cellular phagocytosis (ADCP), phagocytosis rates of MRC-5 fibroblasts were analyzed. MRC-5 cells were loaded with HLA A*0201-binding peptides, either with CMV pp65 peptide (aa495-503) or melanoma gp100 peptide (aa637-645) as a negative control. The peptide-loaded MRC5 cells were then treated with different antibodies at 2 µg/mL: A*0201/pp65-specific IgG1 “C1” antibody and the three peptide-independent antibodies “B6” (anti-HLA A*0201), cetuximab (anti-EGFR) as a positive control or rituximab (anti-CD20) as a negative control. An additional negative control was performed without antibodies. Anti-CD66b FITC-labelled PMNs (1.5 × 105/well) and anti-CD90 APC-labelled MRC-5 cells were incubated together at a ratio of 1:2 at 37 °C. After four hours, cells were harvested from each well and prepared for flow cytometric analysis (Navios EX flow cytometer, Beckman Coulter, Krefeld, Germany), and the proportion of FITC/APC double-positive PMNs, as an indicator of MRC-5 phagocytosis, was determined after doublet exclusion.

### 4.9. Flow Cytometry

After 1 washing cycle with PBS, tumor cells, fibroblasts and blood cells were incubated at RT for 15 min with flag-tagged IgG-format antibodies (5 µg/mL–10 µg/mL) or tetramerized biotinylated Fab antibodies (10 µg/mL). In the case of tetramer Fab staining, tetramers contained PE-coupled streptavidin. In cases where cells were incubated with IgG-format antibodies, the secondary system comprised a biotinylated anti-human Fc antibody, followed by streptavidin-conjugated R-PE (Jackson, West Grove, PA, USA) at a concentration of 1:300 for 15 min at RT. For evaluation of the binding of the IgG-format CMV antibodies to purified NK cells, the secondary system consisted of a mouse anti-Flag antibody (1:200, 15 min at RT, Merck KGaA, Darmstadt, Germany) and a PE-coupled anti-mouse antibody (1:300, 15 min at RT, Jackson, West Grove, PA, USA). After a final washing cycle, cells were ready for evaluation by flow cytometry. To prepare the EDTA sample for flow cytometry, erythrocytes had to be lysed first (BD FACS TM Lysing solution). Analysis of flow cytometric results was done by a BD FACS Canto using the FlowJo™ v10.10 software. Where possible, at least 104 cells were analyzed. When EDTA blood was used, only lymphocytes were gated and analyzed.

The viability of NK cells and the proportion of CD16+ cells were assessed using a Navios EX flow cytometer (Beckman Coulter, Krefeld, Germany).

### 4.10. Cytotoxicity Assays

Primary fibroblasts, T98G, SK-Mel 23, A431 cells, PBMCs of healthy donors and PBMCs of patients with active CMV infection were used as target cells. Due to low lymphocyte counts in most patients, NK cells of healthy volunteers were used as effector cells for ADCC assays. Cells either remained untreated (native), were loaded with control peptides or were loaded with HLA-matched CMV peptides (15 µg/mL, incubation overnight). Cells were either incubated with the corresponding HLA/CMV-specific IgG constructs (1 µg/mL) or remained without treatment. NK effector cells and target cells (4000/well for fibroblasts and 10,000/well using PBMCs) were co-incubated (96-well U-bottom plate, 4 h, 37 °C) using the indicated E/T ratios.

NK cell-mediated ADCC (lysis rate) was assessed by LDH release from target cells. Supernatants were analyzed for their LDH content by ELISA using the Cytotoxicity Detection Kit Plus (Roche/Sigma-Aldrich Chemie GmbH, Taufkirchen, Germany) according to the manufacturer’s instructions. ELISA plates were quantified using a Varioscan Lux (Thermo Scientific, Darmstadt, Germany). Maximum and spontaneous lysis of the respective target cells were used as controls.

ADCC assays were run in triplicate. F-test and two-sided Student’s t-test were used to calculate the significance of differences between lysis rates.

## 5. Conclusions

To summarize, the CMV-specific TCRLs A6, C1 and C7 can be used to detect CMV infection and show therapeutic potential in patients expressing at least one of the three HLA alleles A*0101, A*0201 or B*0702. These antibodies thus significantly expand the potential patient pool for the application of CMV-specific TCRLs.

## Figures and Tables

**Table 1:** Phagocytosis rates of peptide-loaded MRC-5 cells.

Loaded Peptide	Antibody Treatment	Phagocytosis Rate (by PMN in %)
CMV/pp65	no antibody	4.38
	Rituximab (2 µg/mL)	6.66
	C1 (0.5 µg/mL)	16.68
	C1 (2 µg/mL)	22.70
	B6 (2 µg/mL)	27.03
	Cetuximab (2 µg/mL)	24.75
gp100	C1 (0.5 µg/mL)	6.10
	C1 (2 µg/mL)	7.40
	B6 (2 µg/mL)	20.85

**Table 2:** List of CMV-infected patients with at least one matching HLA allele.

Patient ID	Patient HLA Status	CMV Viral Load in pB
CMV1	HLA A*0201	700 IU/mL
CMV2	HLA A*0201	130,000 IU/mL
CMV3	HLA A*0201	2300 IU/mL
CMV4	HLA A*0201	10,000 IU/mL
CMV5	HLA A*01	4400 IU/mL
CMV6	HLA A*01, HLA B*07	4100 IU/mL
CMV7	HLA A*0201	1.5 × 10^6^ IU/mL
CMV8	HLA A*01	2200 IU/mL
CMV9	HLA A*02	1400 IU/mL

**Table 3:** CMV peptides used for loading fibroblasts and lymphocytes.

*HLA allele*	A*0101	A*0201	B*0702
*Allele frequency*	15.10%	26.70%	12.00%
*HCMV peptide*	YSEHPTFTSQY	NLVPMVATV	TPRVTGGGAM
*HCMV protein*	pp65	pp65	pp65

**Table 4:** List of control peptides.

Control Peptides	Aa Sequence	Protein	Matching HLA Allele
peptide 2	VLYDRVLKY	SRP68	A*0101
peptide 3	KIADRFLLY	LIM domain-only protein 4	A*0101
peptide 6	IPNEIIHAL	hnRNP M	B*0702
peptide 7	MPRGVVVTL	E3 ubiquitin-protein ligase HECTD1	B*0702
peptide 10	LPHSSSHWL	Melanocyte protein PMEL	A*0201
peptide 12	SLLMWITQV	NY-ESO-1	A*0201

## Data Availability

All data generated or analyzed during this study will be shared by the corresponding author (moritz.bewarder@uks.eu) upon reasonable request.

## Supplementary Materials

The following supporting information can be downloaded at: https://www.mdpi.com/article/10.3390/ijms252312908/s1.
