# Supplementary material for: Cytomegalovirus-Specific T-Cell-Receptor-like Antibodies Target In Vivo-Infected Human Leukocytes Inducing Natural Killer Cell-Mediated Antibody-Dependent Cellular Cytotoxicity
Source: Int J Mol Sci. 2024 Nov 30;25(23):12908. doi: 10.3390/ijms252312908 (PMC11640865; doi:10.3390/ijms252312908)
Supplement: Supplementary file 1 [file ijms-25-12908-s001.zip › ijms-3295046-supplementary.pdf]

Supplementary Tables

Suppl. Table S1: Controls with no CMV infection and no matching HLA allele

Patient ID	HLA Status	CMV Status
KP4	Neg. for HLA A*0201, A*0101 and B*0702	negative
KP5	Neg. for HLA A*0201, A*0101 and B*0702	negative
KP6	Neg. for HLA A*0201, A*0101 and B*0702	negative

Suppl. Table S2: Controls with no CMV infection but with matching HLA allele

Patient ID	HLA Status	CMV Status
KP1	HLA A*0201	negative
KP3	HLA A*0201<br>HLA B*0702	negative
KP8	HLA A*0101	negative

Suppl. Table S3: Controls with CMV infection but no matching HLA allele

Patient ID	HLA Status	CMV Status
KP10	Neg. for HLA A*0201, A*0101 and B*0702	2700 IU/ml
KP11	Neg. for HLA A*0201, A*0101 and B*0702	2200 IU/ml
KP12	Neg. for HLA A*0201, A*0101 and B*0702	9900 IU/ml
KP13	Neg. for HLA A*0201, A*0101 and B*0702	2600 IU/ml
KP14	Neg. for HLA A*0201, A*0101 and B*0702	1500 IU/ml
KP15	Neg. for HLA A*0201, A*0101 and B*0702	1000 IU/ml
KP16	Neg. for HLA A*0201, A*0101 and B*0702	1500 IU/ml
KP17	Neg. for HLA A*0201, A*0101 and B*0702	10.500 IU/ml
KP18	Neg. for HLA A*0201, A*0101 and B*0702	8000 IU/ml

Supplementary Figures

Suppl. Figure S1

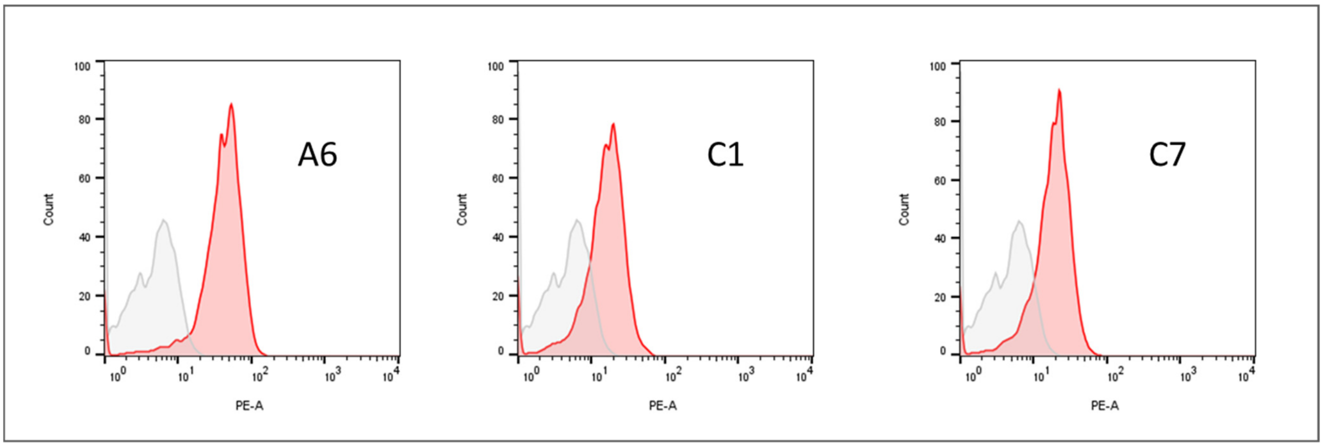

Supplemental Figure S1: Binding of anti-CMV antibodies to NK cells

The three CMV-specific antibodies in IgG format—A6, C1 and C7—were analyzed by flow cytometry and showed strong binding to isolated NK cells. Anti-Flag followed by anti-mouse PE antibodies were used as a secondary system. NK cells were isolated from PBMCs by magnetic depletion of all non-NK cells using the CD56+/CD16+ human NK Cell Isolation Kit (Miltenyi Biotech GmbH, Bergisch Gladbach, Germany).

Grey: Secondary antibody only, Red: CMV AB.

Suppl. Figure S2

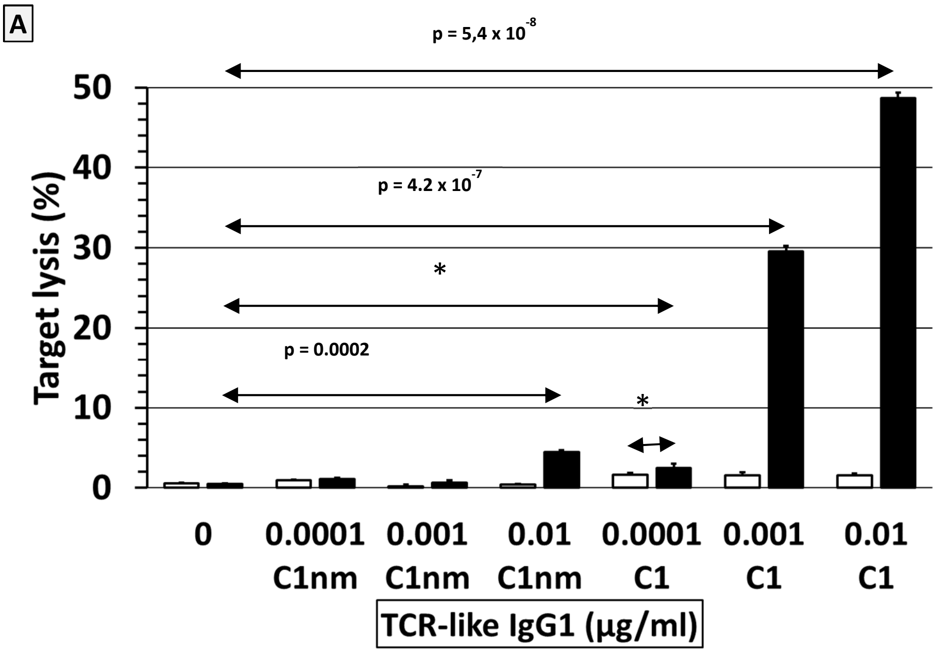

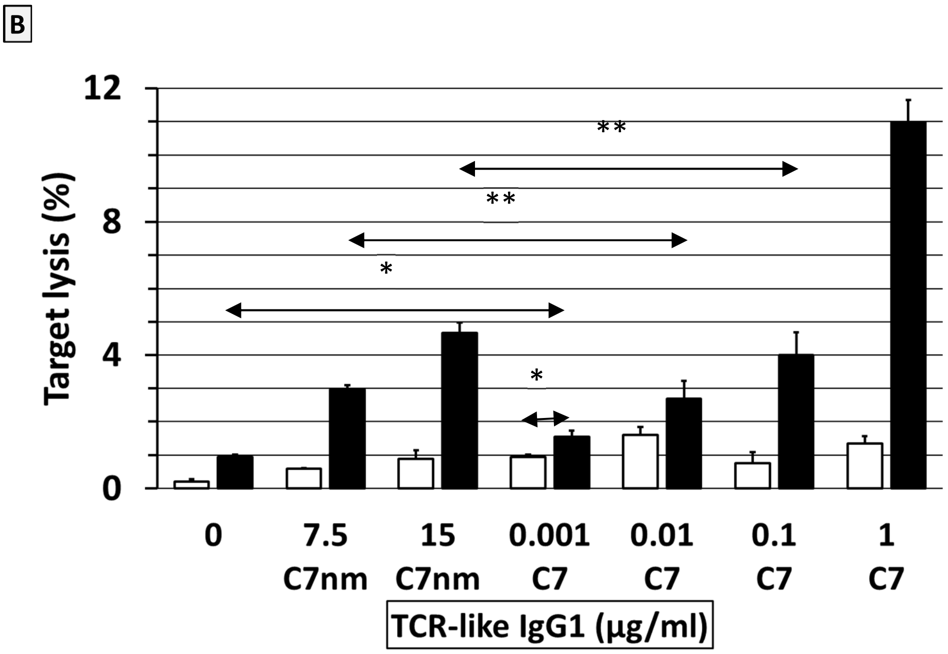

Supplemental Figure S2: Improved ADCC after Fc modification of C1 and C7.

Fibroblasts and effector NK cells were obtained from the same donor. Fibroblasts were loaded with either a non-CMV peptide (white columns) or a CMV/pp65 epitope (black columns), each matching the MHC I type under investigation. **(A)** The unmodified A*0201/pp65(aa495-503)-specific IgG1 antibody "C1nm" only induced weak NK-mediated ADCC at 0.01 µg/ml. After modification, the TCR-like antibody "C1" induced significant ADCC starting at 0.001 µg/ml (*) and an E/T ratio of 5:1. **(B)** Without Fc modification, the B*0702/pp65(417-426)-specific antibody "C7nm" induced significant ADCC of target cells only at very high doses of 7.5 and 15 µg/ml. Fc modification resulted in significantly improved efficacy, and ADCC induced by C7 reached 4% and 11% at 0.1 and 1 µg/ml at an E/T ratio of 10:1.

Suppl. Figure S3

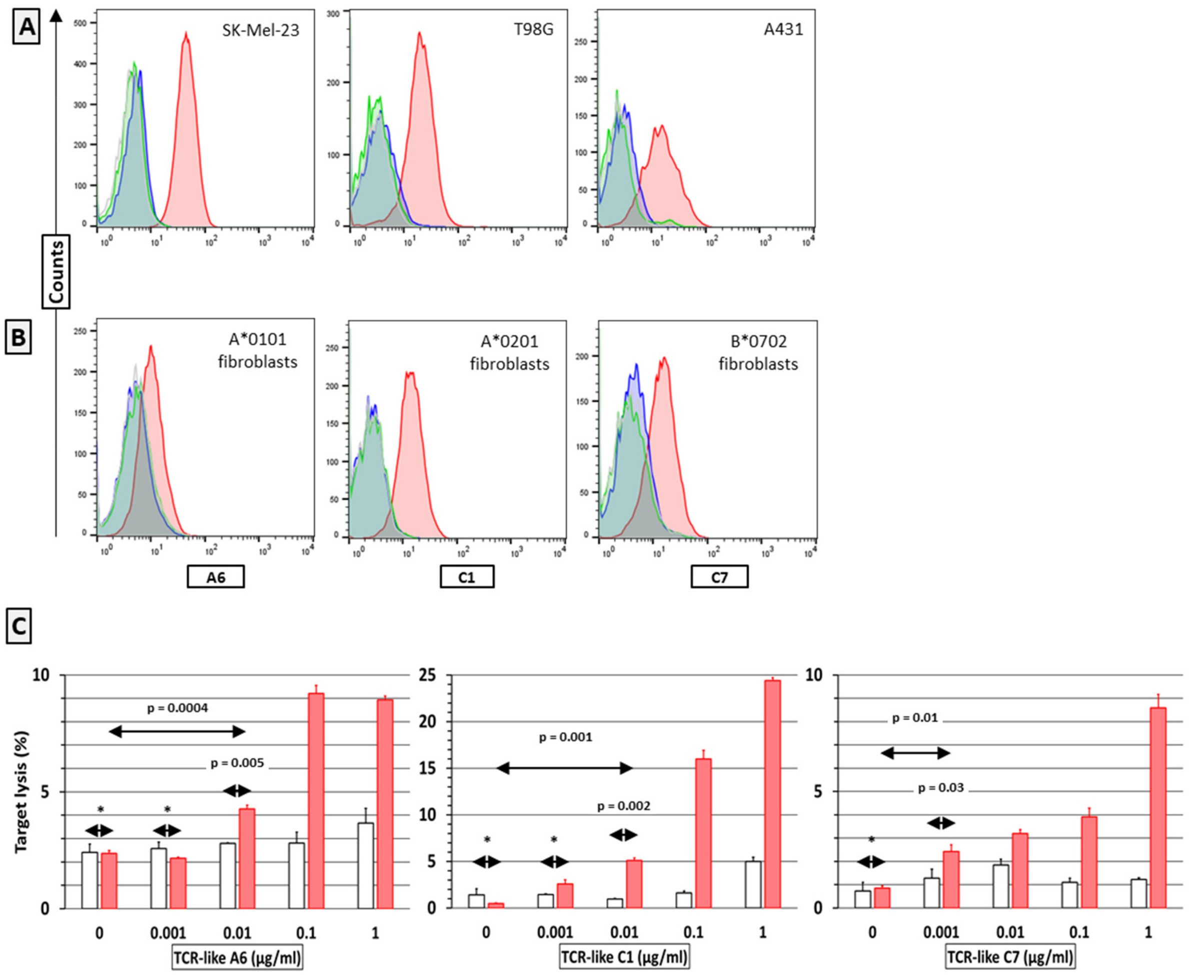

Supplemental Figure S3: Effect of CMV-specific TCR-like antibodies on CMV peptide-loaded cells.

The CMV-specific TCR-like antibodies A6, C1 and C7 were tested for binding to CMV peptide-loaded tumor cells and primary fibroblasts and for their potential to induce ADCC in CMV peptide-loaded fibroblasts after the addition of NK cells. **(A)** CMV-specific antibodies were tested for binding to CMV peptide-pulsed tumor cells with matching HLA alleles and showed robust binding. **(B)** A6, C1 and C7 demonstrated binding to CMV peptide-pulsed primary fibroblasts expressing matching HLA alleles. **(C)** After addition of purified NK cells, CMV-specific TCR-like antibodies induced specific cytotoxic effects in CMV peptide-loaded fibroblasts.
Grey: Secondary antibody only, Green: CMV peptide only, Blue: CMV AB only, Red: CMV AB on peptide-loaded cells.

Suppl. Figure S4

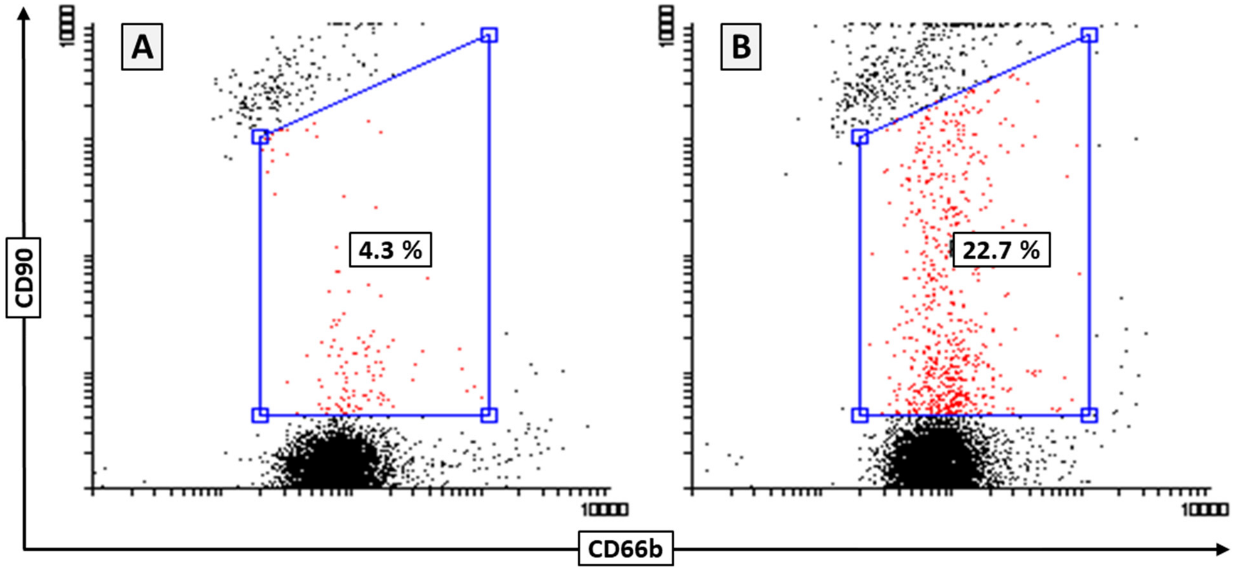

Supplemental Figure S4: ADCP driven by polymorphonuclear neutrophils.

To test the potential of the MHC-I/CMV-specific IgG1 constructs to induce phagocytosis of CMV-infected host cells into PMNs, CMV peptide-loaded (pp65 epitope aa495-503) MRC-5 fibroblasts were used as target cells. MRC-5 cells were treated with different antibodies and incubated with PMNs. For flow cytometric assessment of phagocytosis rates, PMNs were fluorescence-labeled with anti-CD66b antibodies and MRC-5 cells were labeled with anti-CD90 antibodies. The phagocytosis rate was measured as the proportion of CD66b+/CD90+ PMNs after four hours of incubation. Exemplary illustrations of the analysis are shown in **(A)** without antibody treatment as negative control and **(B)** after treatment with 2 µg/ml of the HLA-A*0201/pp65(aa495-503)-specific IgG antibody C1.

Suppl. Figure 5

A

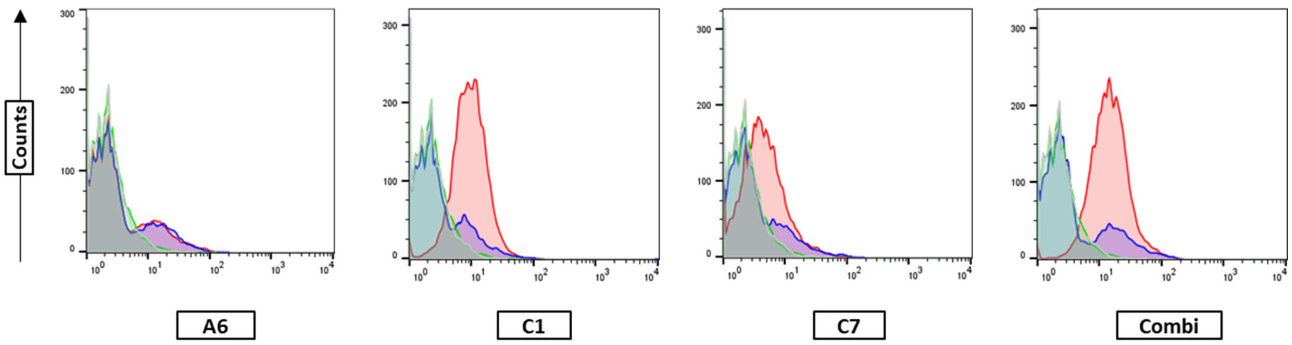

B

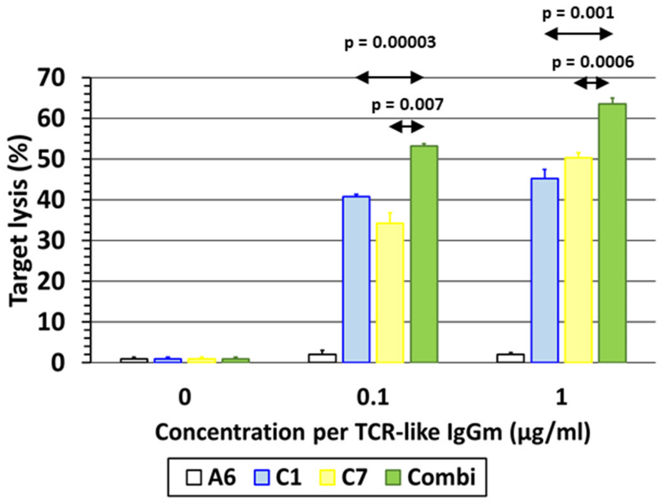

Supplemental Figure S5: Application of CMV-specific antibodies as an antibody pool.

(A) Lymphocytes positive for A*0201 and B*0702 and negative for A*0101 were stained with the CMV-specific antibodies C1, C7 and A6 separately and in combination. A6 showed no binding, while C1 and C7 both demonstrated binding to CMV peptide-loaded lymphocytes. When lymphocytes are stained with a mix of A6, C1 and C7, MFI increases to add up the MFIs generated by C1 and C7.
Grey: Secondary antibody only, Green: CMV peptide only, Blue: CMV AB only, Red: CMV AB on peptide-loaded cells.

(B) Cytotoxicity assays were performed accordingly. The addition of A6 did not result in specific lysis of CMV peptide-loaded lymphocytes. When used individually, the addition of C1 and C7 resulted in specific lysis rates of 40% and 35% at a concentration of 0.1 µg/ml, going up to 45% and 50% at 1 µg/ml, respectively. When all three CMV-specific antibodies were used in combination on CMV peptide-loaded lymphocytes, specific lysis rates increased to 53% at 0.1 µg/ml and 64% at 1 µg/ml (Suppl. Fig. 5 B).

Suppl. Figure S6

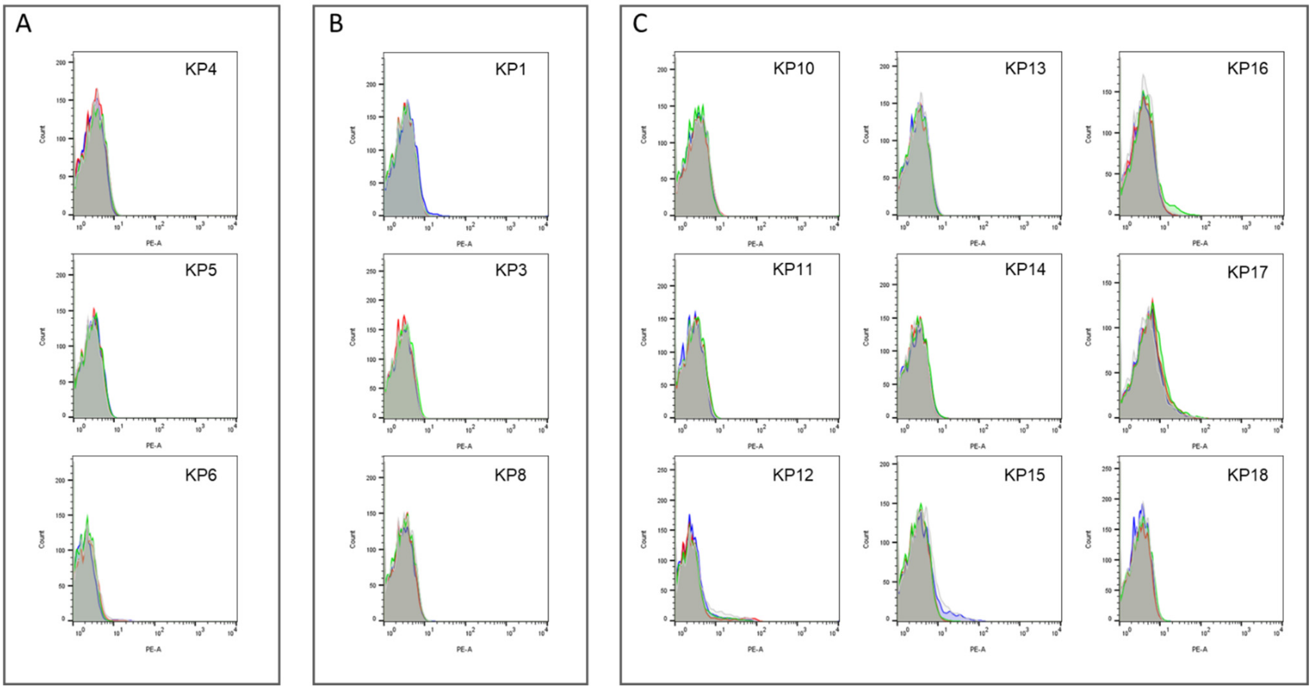

Supplemental Figure S6: A6, C1 and C7 on control lymphocytes.

Peripheral blood samples of healthy controls and control patients were incubated at RT for 15 min with tetramerized biotinylated Fab antibodies at a concentration of 10 µg/mL. Tetramers contain PE-coupled streptavidin for detection by flow cytometry. As controls, blood from healthy donors without CMV infection and no matching HLA status **(A)**, blood from healthy donors without CMV infection with at least one matching HLA allele **(B)** and blood from control patients with CMV infection but no matching HLA allele **(C)** were used. A6, C1 and C7 showed no binding to lymphocytes of controls **(A-C)**.

Grey: negative control, Green: A6 antibody, Red: C1 antibody, Blue: C7 antibody.
